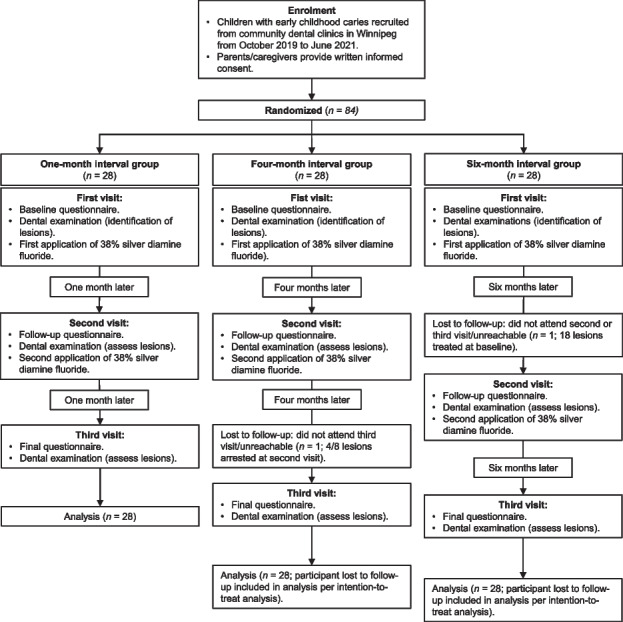# Correction: An open‑label, parallel‑group, randomized clinical trial of different silver diamine fluoride application intervals to arrest dental caries

**DOI:** 10.1186/s12903-024-05024-9

**Published:** 2024-11-07

**Authors:** Robert J. Schroth, Sukeerat Bajwa, Victor H. K. Lee, Betty‑Anne Mittermuller, Sarbjeet Singh, Vivianne Cruz de Jesus, Mary Bertone, Prashen Chelikani

**Affiliations:** 1 Department of Preventive Dental Science, Dr. Gerald Niznick College of Dentistry, Rady Faculty of Health Sciences, University of Manitoba, 507-715 McDermot Avenue, Winnipeg, MB R3E 3P4 Canada; 2 Department of Pediatrics and Child Health, Max Rady College of Medicine, Rady Faculty of Health Sciences, University of Manitoba, Winnipeg, MB Canada; 3 Children’s Hospital Research Institute of Manitoba, Winnipeg, MB Canada; 4 Shared Health Inc, Winnipeg, MB Canada; 5 School of Dental Hygiene, Dr. Gerald Niznick College of Dentistry, Rady Faculty of Health Sciences, University of Manitoba, Winnipeg, MB Canada; 6 Department of Oral Biology, Dr. Gerald Niznick College of Dentistry, Rady Faculty of Health Sciences, University of Manitoba, Winnipeg, MB Canada

**Correction** **:** **BMC Oral Health 24, 1036 (2024)**

**https://doi.org/10.1186/s12903-024-04791-9**

Following the publication of the original article [1], the author would like to correct the typo in Figure 1. The flow diagram incorrectly states that at the third visit a second application of SDF/FV was performed. This should not be there. The incorrect and correct Figure 1 are shown in this correction article.

**Incorrect Figure 1** 
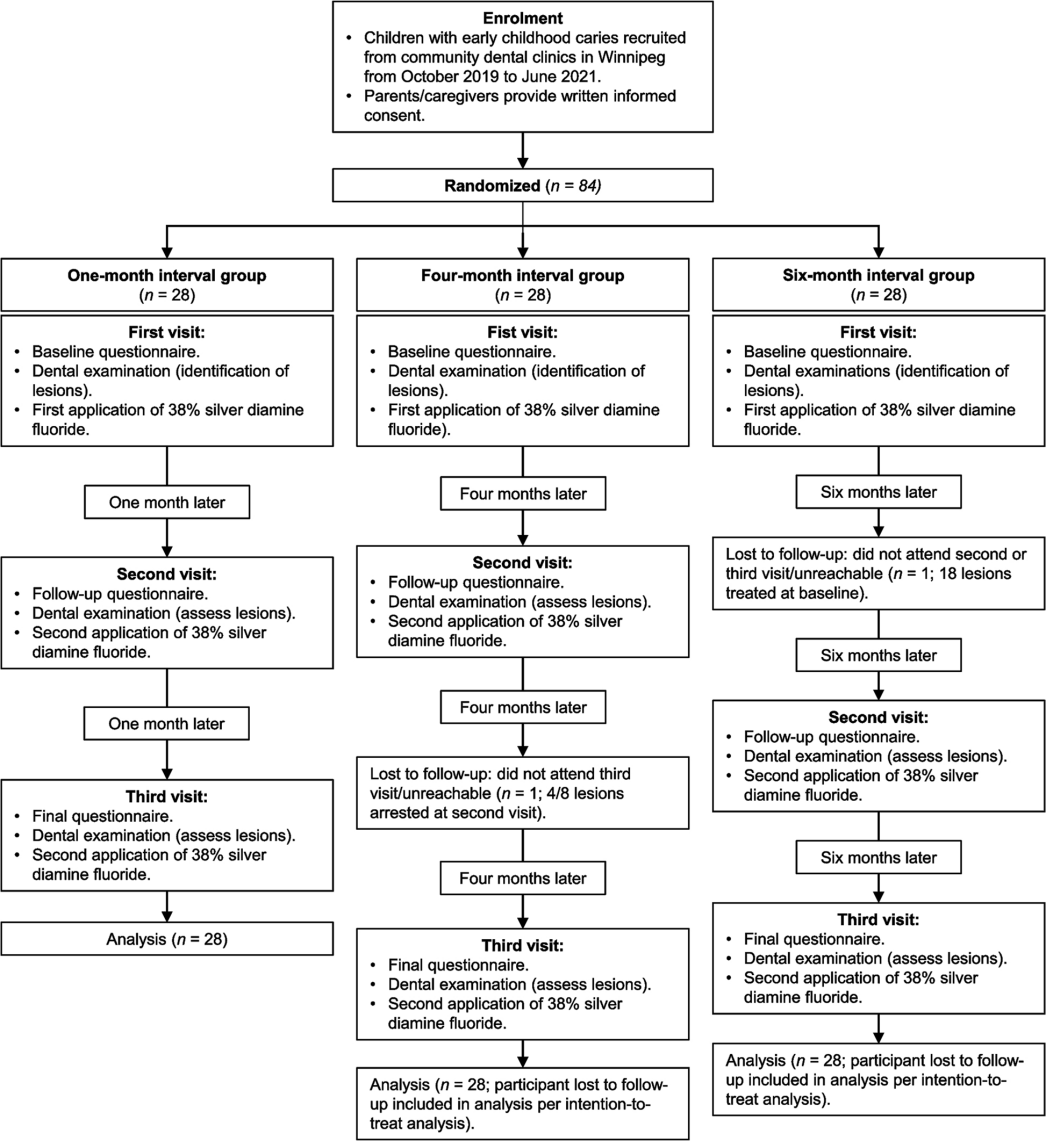


**Correct Figure 1**